# New *Burst-Oscillation Mode* in Paced One-Dimensional Excitable Systems

**DOI:** 10.3389/fphys.2022.854887

**Published:** 2022-03-23

**Authors:** Zhao Lei, Jiajing Liu, Yaru Zhao, Fei Liu, Yu Qian, Zhigang Zheng

**Affiliations:** ^1^College of Physics and Optoelectronic Technology, Baoji University of Arts and Sciences, Baoji, China; ^2^Advanced Titanium Alloys and Functional Coatings Cooperative Innovation Center, Baoji, China; ^3^Institute of Systems Science, Huaqiao University, Xiamen, China; ^4^School of Mathematical Sciences, Huaqiao University, Quanzhou, China; ^5^College of Information Science and Engineering, Huaqiao University, Xiamen, China

**Keywords:** *burst-oscillation mode*, excitable systems, nonlinear dynamcs, spikes, complex networks

## Abstract

A new type of *burst-oscillation mode* (BOM) is reported for the first time, by extensively investigating the response dynamics of a one-dimensional (1D) paced excitable system with unidirectional coupling. The BOM state is an alternating transition between two distinct phases, i.e., the phase with multiple short spikes and the phase with a long interval. The realizable region and the unrealizable region for the evolution of BOM are identified, which is determined by the initial pulse number in the system. It is revealed that, in the realizable region, the initial inhomogeneous BOM will eventually evolve to the homogeneously distributed *spike-oscillation mode* (SOM), while it can maintain in the unrealizable region. Furthermore, several dynamical features of BOM and SOM are theoretically predicted and have been verified in numerical simulations. The mechanisms of the emergence of BOM are discussed in detail. It is revealed that three key factors, i.e., the linking time, the system length, and the local dynamics, can effectively modulate the pattern of BOM. Moreover, the suitable parameter region of the external pacing (*A, f*) that can produce the new type of BOM, has been explicitly revealed. These results may facilitate a deeper understanding of bursts in nature and will have a useful impact in related fields.

## Introduction

Burst oscillation is a compound dynamical behavior alternating between the active phase and the silent phase (Desroches et al., 2012; Fallah, 2016). In the active phase, the system shows rapid and successive spikes with a relatively large-amplitude. While in the silent phase, the variables exhibit small-amplitude vibrations, which can be considered as the quiescent state. Burst oscillation behaviors can be extensively observed in a variety of systems, ranging from physical, chemical, biological, and neuronal systems (Decroly and Goldbeter, 1987; Sherman et al., 1988; Cymbalyuk et al., 2002; DeShazer et al., 2003; Xie et al., 2003; Russella et al., 2010; Marino et al., 2011). For example, Sherman et al. revealed the emergence of organized bursting in clusters of pancreatic beta-cells induced by channel sharing (Sherman et al., 1988). Cymbalyuk et al. reported the bursting in leech heart interneurons (Cymbalyuk et al., 2002). DeShazer et al. studied the bursting dynamics of a fiber laser with an injected signal (DeShazer et al., 2003). Xie et al. (2003) revealed the parabolic bursting induced by veratridine in rat injured sciatic nerves. In the past decades, the topic of burst oscillation is one of the most important interdisciplinary issues in nonlinear science and biology. It has been found that burst oscillation plays a key role in determining specific physiological processes. Steriade et al. (1993) exposed the thalamocortical burst oscillations in the sleeping and aroused brain. Lisman discovered that bursts as a unit of neural information can make synapses reliable in signal transmission (Lisman, 1997). Reinagel et al. (1999) revealed the encoding of visual information by the bursts of the dorsal lateral geniculate nucleus of the thalamus. Izhikevich et al. (2003) found that bursts of action potentials might provide effective mechanisms for selective communication between neurons.

Theoretically, burst oscillations can be usually observed in single dynamical systems with distinct timescales, and have been extensively studied as the fast-slow Hodgkin-Huxley model was proposed (Hodgkin and Huxley, 1952). Since the fast-slow analysis method was proposed by Rinzel in 1985, which can effectively expose the formation and the mechanism of burst oscillation, great achievements have been made in this field. Distinct modes of burst oscillation were identified in different kinds of theoretical models and the corresponding mechanisms were further revealed (de Vries, 1998; Perc and Marhl, 2003; Zhang et al., 2007; Han and Bi, 2011; Yang and Hao, 2014; Vijay et al., 2019; Ma et al., 2021; Qian et al., 2021b). For example, Vries discovered multiple bifurcations of bursting oscillations in a polynomial model (de Vries, 1998). Perc and Marhl discussed diverse types of bursting calcium oscillations in non-excitable cells (Perc and Marhl, 2003). Vijay et al. investigated different transitions of bursting and mixed-mode oscillations in the Liénard system with external sinusoidal forcing (Vijay et al., 2019). Ma et al. reported the complex bursting dynamics in a van der Pol-Mathieu-Duffing system with multiple-frequency slow-varying excitations and revealed the pulse-shaped explosion as a special route to bursting oscillations (Ma et al., 2021). Most of these contributions are devoted to the burst oscillations in the single systems. However, less dedications are made in the complex systems with multi-units.

Since the small-world (Watts and Strogatz, 1998) and scale-free (Barabási and Albert, 1999) network models were, respectively, proposed by Watts and Barabási, the problems of complex systems have become the central topics under investigation due to their extensive applications, among which the interplay between structure and dynamics is one of the most important subjects. Tremendous contributions are achieved in this aspect, and peoples have confirmed that network structure does play a key role in determining the spatiotemporal dynamics of the system (Zhou et al., 2007; Wang et al., 2008; Bogaard et al., 2009; Pernice et al., 2011; Xu et al., 2013; Gonzalez et al., 2014; Hütt et al., 2014; Jovanović and Rotter, 2016). For example, Zhou et al. discussed the structure-function relationship in complex brain networks expressed by hierarchical synchronization (Zhou et al., 2007). Pernice et al. studied how structure determines correlations in neuronal networks (Pernice et al., 2011). Xu et al. investigated the control of self-sustained spiking activity by adding or removing one network link (Xu et al., 2013). Hütt et al. reviewed the network-guided pattern formation of neural dynamics (Hütt et al., 2014).

Excitable dynamics is popular, it can implement a perfect spiking as stimulated by a supra-threshold excitation. In the last decades, excitable dynamics has been widely exploited to study the issues of oscillation in the interdiscipline of physics and biological science. Network structure determined oscillation modes in diverse excitable complex networks are the hot topic in this field, and lots of dedications have been accomplished (Qian et al., 2010a,b, 2019, 2020, 2021a; Qian, 2014).

It is well known that the dynamical behaviors of electrical activities in neurons are complicated. It had been exposed that, with the change of intrinsic parameters or external environment, a single neuron can exhibit multiple modes of electrical activities, such as spiking, bursting, and even chaos oscillations. Different from the abundant dynamical behaviors of neurons, a single excitable cell can only perform spiking dynamics. To produce burst oscillations, the excitable complex network is a feasible way to achieve this goal. To our knowledge, this issue has not been extensively discussed. Furthermore, it is also very interesting to expose the topological structure conditions that can enable the emergence of burst oscillations in excitable complex networks and the corresponding bursting dynamical features.

The edges linking neurons in neuronal networks and brain systems are extremely complicated. This definitely constitutes complex network topologies, such as small-world or scale-free structures. However, among these complex connections, there exist huge numbers of one-dimensional (1D) topological rings. More importantly, as we have confirmed, excitable waves can propagate unidirectional along these 1D structures to form 1D unidirectional Winfree loops as the sources maintaining the oscillations in excitable complex networks (Qian et al., 2010a). So the investigation of the spatiotemporal dynamics in 1D unidirectionally excitable system is of great importance. Furthermore, the 1D system is also the simplest structure, which is conducive to discuss and analyze. The conclusions obtained from the 1D system are also instructive for understanding the phenomena emerging in complex systems. In this paper, the simplest model of an excitable complex network, i.e., the 1D excitable system with external periodic pacing, is proposed. The spatiotemporal dynamics with diverse pacings on this straightforward network model are extensively studied, and abundant response behaviors are exposed, among which a new type of *burst-oscillation mode* (BOM) is revealed for the first time.

The remainder of the paper is organized as follows. We first introduce the mathematical model and the response dynamics of the 1D paced excitable system and then discuss the emergence of BOM and its dynamic features. We further reveal the determinants for the BOM and give the effective parameter region of external pacing in producing the BOM.

## Mathematical Model and Response Dynamics

The 1D unidirectional excitable chain with periodic pacing is first constructed. The Bär-Eiswirth (Bär and Eiswirth, 1993) model is adopted as the local excitable dynamics.

This excitable model has typical intrinsic simplicity and low dimensionality, it possesses the key property of excitable dynamics and can imitate the main dynamical features of complicated neuron dynamics with essentially lower computational costs. Importantly, several important contributions are achieved based on this model (McGraw and Menzinger, 2011; Gu et al., 2013; Mi et al., 2013), among which Mi et al. revealed the long-period rhythmic synchronous firing in a Barabási-Albert-like scale-free network and proposed a Hebbian learning mechanism leading to topologically similar neuronal networks as the basis for the memorization of information encoded in long temporal intervals (Mi et al., 2013).

Here, an external periodic pacing *Asin*(2π*ft*) with *A* and *f* being the amplitude and frequency is introduced, which is applied to the fast variable of the first node *u*_1_(*t*). The evolution of the paced excitable unidirectional chain satisfies the following equations


(1) $$\frac{\text{d}u_{i}(t)}{\text{d}t}=\{\begin{array}{cc} \frac{1}{\varepsilon }g(u_{i},v_{i})+Asin(2\pi ft) & \text{for }i=1,\\ \frac{1}{\varepsilon }g(u_{i},v_{i})+D[u_{i-1}(t)-u_{i}(t)] & \text{for }i=2,\ldots ,L, \end{array}$$

(2) $$\begin{array}{l} \frac{\text{d}v_{i}\left(t\right)}{\text{d}t}=F\left[u_{i}\left(t\right)\right]-v_{i}\left(t\right), \end{array}$$

where


(3) $$\begin{array}{l} g\left(u,v\right)=u\left(1-u\right)\left(u-\frac{v+b}{a}\right). \end{array}$$

Here *F*(*u*) is a piecewise function with the following form


(4) $$F(u)=\{\begin{array}{cc} 0 & u<\frac{1}{3},\\ 1-6.75u{\left(u-1\right)}^{2} & \frac{1}{3}\leq u\leq 1,\\ 1 & u>1. \end{array}$$

In the above equations, the subscript *i* (*i* = 1, 2, …, *L*) denotes the position of the excitable node, where *L* is the length of the system. The variables *u*_*i*_ and *v*_*i*_ are the fast and slow variables of the *i*th cell, respectively. The symbols *a*, *b*, and ε are three dimensionless parameters that can effectively control the local dynamics of the system. *D* is the coupling strength, which describes the action intensity between linking neighbors. For a suitable set of parameters, e.g., *a* = 0.84, *b* = 0.07, ε = 0.04, *D* = 1.0, and *L* = 500 (this parameter selection will be adopted throughout this paper if there is no special explanation), a typical 1D paced unidirectional excitable chain can be built.

Based on the above model, we can construct the 1D paced unidirectional excitable ring by introducing a one-way link from the end node *i* = *L* to the head node *i* = 1 of Equation (1). The linking time *T*_Link_ is introduced and defined as the time we add this additional unidirectional connection. When *t* ≥ *T*_Link_, Equation (1) can be transformed into


(5) $$\begin{array}{l} \frac{\text{d}u_{i}\left(t\right)}{\text{d}t}=\frac{1}{\varepsilon }g\left(u_{i},v_{i}\right)+\delta_{i,1}Asin\left(2\pi ft\right)+D\left[u_{i-1}\left(t\right)-u_{i}\left(t\right)\right]\\ \text{for }i=1,\ldots ,L. \end{array}$$

Here δ_*i,j*_ is a function, which is defined as δ_*i,j*_ = 1 if *i* = *j*, and δ_*i,j*_ = 0 otherwise.

In the following numerical studies, the above mathematical model is integrated by the forward Euler method with the time step Δ*t* = 0.02. Here, we should mention that the same results can also be obtained for smaller time steps. The initial values of the fast and slow variables of each node are set as *u*_*i*_(*t* = 0) = 0 and *v*_*i*_(*t* = 0) = 0. This means that, initially, the 1D system is in the homogeneous rest state.

**Figure 1:**
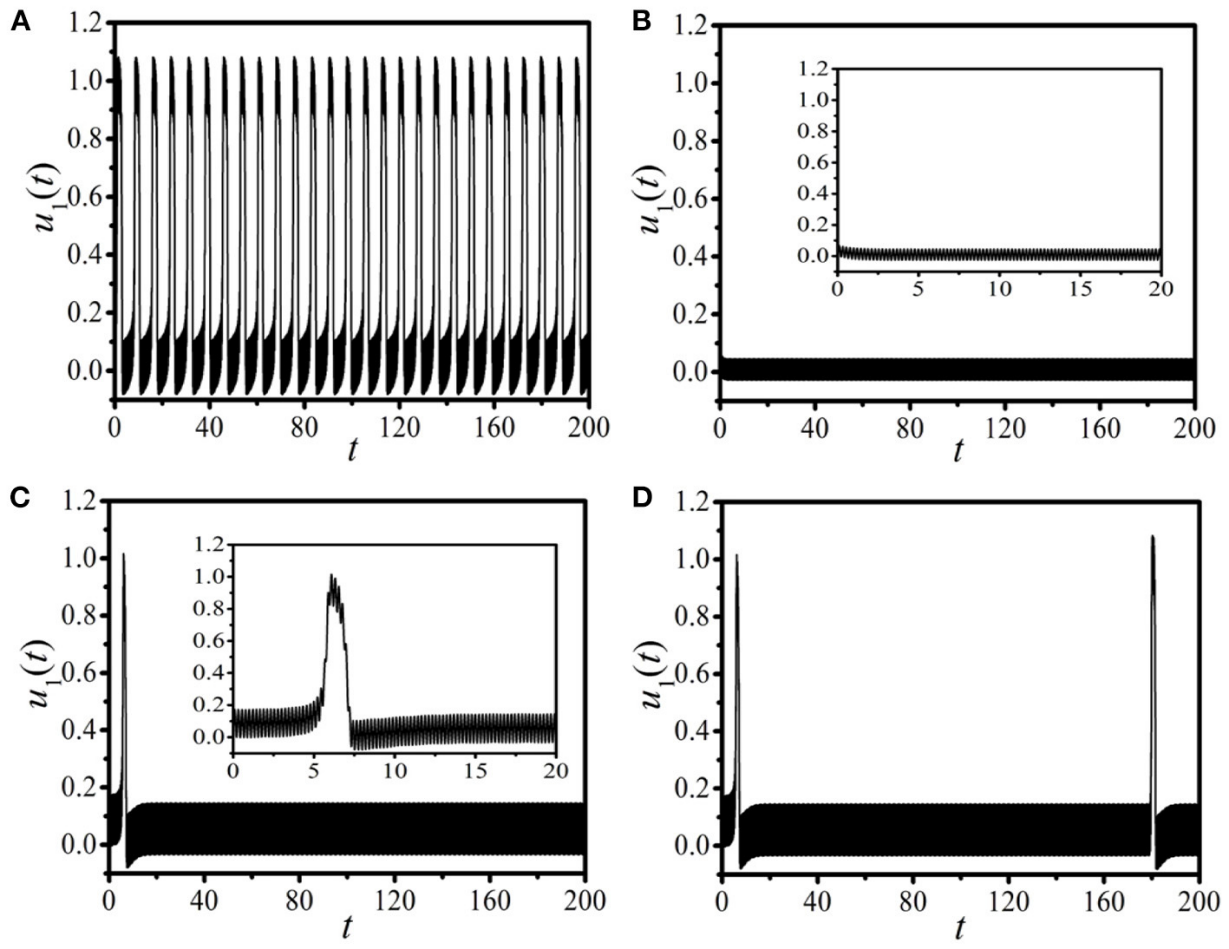
The response dynamics of *u*_1_(*t*) in the one-dimensional (1D) periodically paced unidirectional excitable chain **(A,B)** and ring **(C,D)**. The external periodic pacing *Asin*(2π*ft*) is applied on *u*_1_. **(A,B)**: The distinct response dynamics in the chain with two specific sets of pacing parameters: the supra-threshold spiking with pacing (*A,f*) = (2.5, 4.5) **(A)** and the sub-threshold vibration with pacing (*A,f*) = (1.0, 4.5) **(B)**. **(C,D)**: The distinct response dynamics in the ring with the same pacing parameter as **(A)** and different boundary conditions: the transition from the supra-threshold spiking to the sub-threshold vibrations with the fixed boundary condition *u*_*L*_(*t*) = *v*_*L*_(*t*) = 0 **(C)**, and the *burst-oscillation mode* (BOM) with the periodic boundary condition *u*_*i*+*L*_(*t*) = *u*_*i*_(*t*), *v*_*i*+*L*_(*t*) = *v*_*i*_(*t*) **(D)**. The insets in **(B)** and **(C)** show the local amplification in the time interval *t* ∈ [0,20].

Let us first study the dynamics of the unidirectional excitable chain under the drive of the periodic pacings with different amplitudes and frequencies. Figures 1A,B, respectively, display the response dynamics of *u*_1_(*t*) for the 1D unidirectional excitable chain paced by two specific sets of pacing parameters (*A,f*) = (2.5, 4.5) (Figure 1A) and (*A,f*) = (1.0, 4.5) (Figure 1B). It is shown that, for different pacings, two distinct oscillation modes with discrepant response amplitudes are observed. Figure 1A shows a typical oscillation mode of the excitable dynamics with a large amplitude, which is identified as the supra-threshold spiking. On the contrary, an abnormal oscillation of excitable dynamics with a small amplitude is detected in Figure 1B, and we identify this kind of oscillation mode as the sub-threshold vibration. It is exposed in Figures 1A,B that, with different pacings, distinct oscillation modes with large or small amplitudes can be gained. This means that the external pacing is an effective factor to control the response mode of the paced excitable dynamics.

Now, we discuss the response dynamics in the 1D paced unidirectional excitable ring instead of the chain topology. Figures 1C,D, respectively, display the response results with the same pacing parameter as Figure 1A [i.e., (*A,f*) = (2.5, 4.5)] and different boundary conditions. The linking time is selected as *T*_Link_ = 0. Figure 1C shows the response trajectory for the fixed boundary condition *u*_*L*_(*t*) = *v*_*L*_(*t*) = 0. Based on the results shown in Figures 1A,C we can find that, by introducing a unidirectional link on the paced 1*st* node and adding the fixed boundary condition, the initial supra-threshold oscillation is suppressed, and the response dynamics changes from the supra-threshold spiking to the sub-threshold vibration. Figure 1D exhibits distinct response dynamics attained with the periodic boundary condition *u*_*i*+*L*_(*t*) = *u*_*i*_(*t*), *v*_*i*+*L*_(*t*) = *v*_*i*_(*t*), where the alternate oscillation between the supra-threshold spiking and the sub-threshold vibration is exposed. This new type of oscillation mode is absolutely different from the response dynamics observed in Figures 1A–C, which is similar to the bursts observed in the dynamical systems with distinct timescales. This kind of new oscillation mode emerged in the 1D paced unidirectional excitable ring can be called the *BOM*. Based on these discussions we can conclude that, the network structure and the boundary conditions are two key points to regulate the response dynamics of the paced excitable dynamics.

## The BOM and Its Dynamical Features

Diverse response dynamical behaviors have been identified in Figure 1 in the 1D paced unidirectional excitable chain/ring with appropriate periodic pacings and different boundary conditions. The finding of the new type of BOM is very interesting. It is well known that typical dynamical behavior of burst is the existence of multiple spikings in one oscillation period. However, the BOM shown in Figure 1D only has one pulse in a period. It is thus important whether there exists the BOM with multiple pulses in this model.

**Figure 2:**
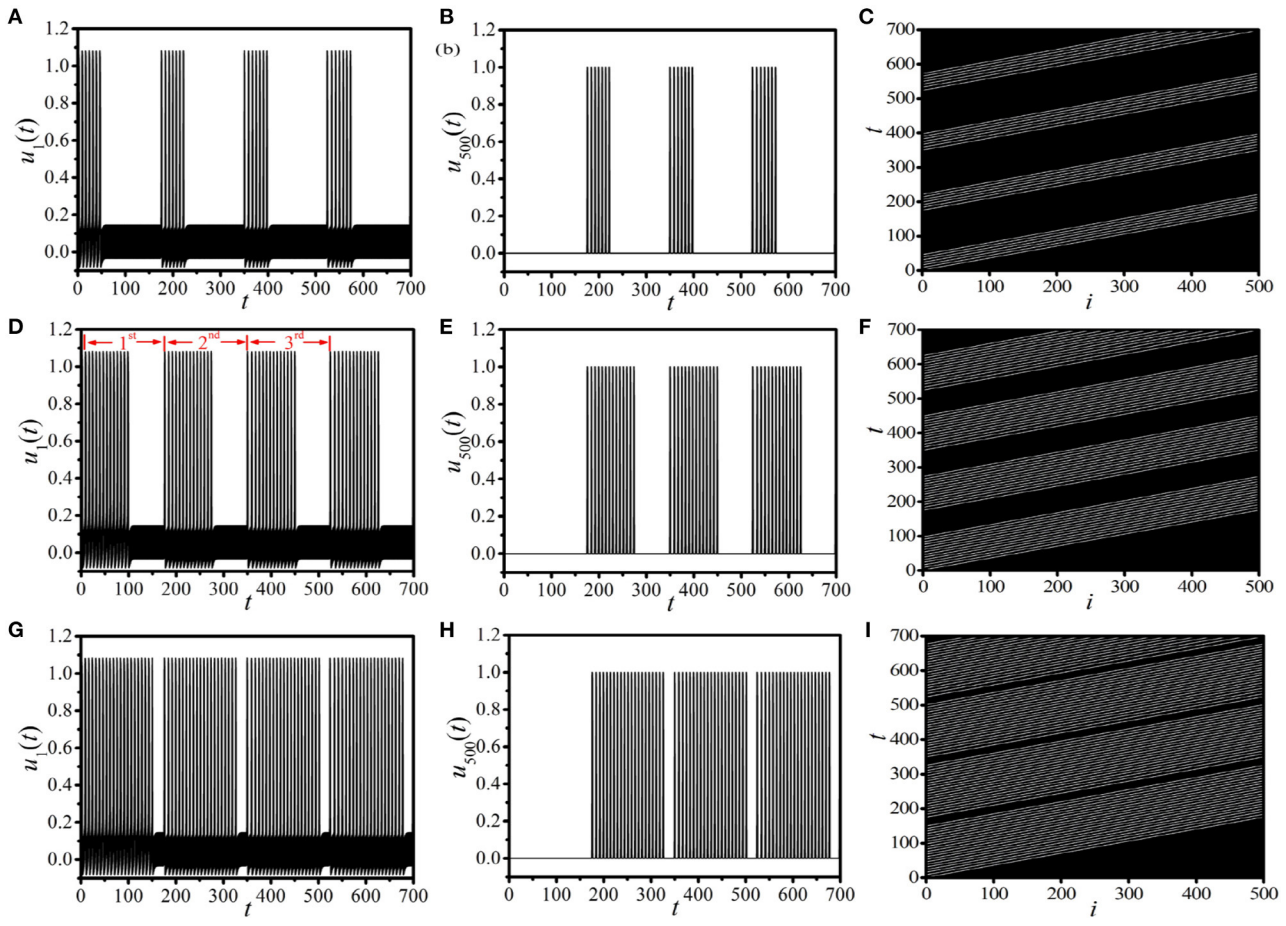
(Color online) Diverse BOM patterns in the 1D paced unidirectional excitable ring with different linking times *T*_Link_. **(A–C)**: *T*_Link_ = 50; **(D–F)**: *T*_Link_ = 100; **(G–I)**: *T*_Link_ = 150. The left, middle, and right columns, respectively, present the response dynamics of *u*_1_(*t*), *u*_500_(*t*), and the response spatiotemporal pattern. The labels 1*st*, 2*nd*, and 3*rd* in **(D)** denote the first three burst periods.

As shown in Figures 1A,C, the supra-threshold spiking will be suppressed immediately as the unidirectional link from the end node *i* = *L* to the head node *i* = 1 is added. It is possible to observe the BOM with multiple pulses by modulating the time of constructing the 1D unidirectional ring. Figure 2 shows the interesting multiple-burst behavior in 1D periodically paced unidirectional excitable ring by modulating the linking times *T*_Link_, where the response dynamics of *u*_1_(*t*) [left column (*a*), (*d*), (*g*)] and *u*_500_(*t*) [middle column (*b*), (*e*), (*h*)], and the response spatiotemporal pattern [right column (*c*), (*f*), (*i*)] are presented. The periodic pacing is selected as (*A,f*) = (2.5, 4.5) (the same as Figure 1A), and is acted on *u*_1_. Figures 2A–C show the BOM with *T*_Link_ = 50, where a bundle of 7 pulses in one burst period are obtained. Importantly, this new type of BOM can propagate along the ring persistently (see the spatiotemporal pattern shown in Figure 2C). As *T*_Link_ is increased (as shown in Figures 2D–F for *T*_Link_ = 100), the pulse number in one burst period *N*_Pulse_ increases remarkably (*N*_Pulse_ = 14) and the interval time between two successive burst periods *T*_Interval_ decreases. The labels 1*st*, 2*nd*, and 3*rd* in Figure 2D, respectively, denote the first three burst periods of BOM. When the linking time increases to *T*_Link_ = 150 (corresponding to Figures 2G–I), *N*_Pulse_ becomes larger, and *T*_Interval_ becomes remarkably shorter. These results indicate that the linking time is a key factor in determining the pattern of BOM in the 1D paced unidirectional excitable ring, especially the pulse number in one burst period and the interval time between two successive burst periods. By adjusting the linking time, diverse BOMs with different patterns can be achieved.

**Figure 3:**
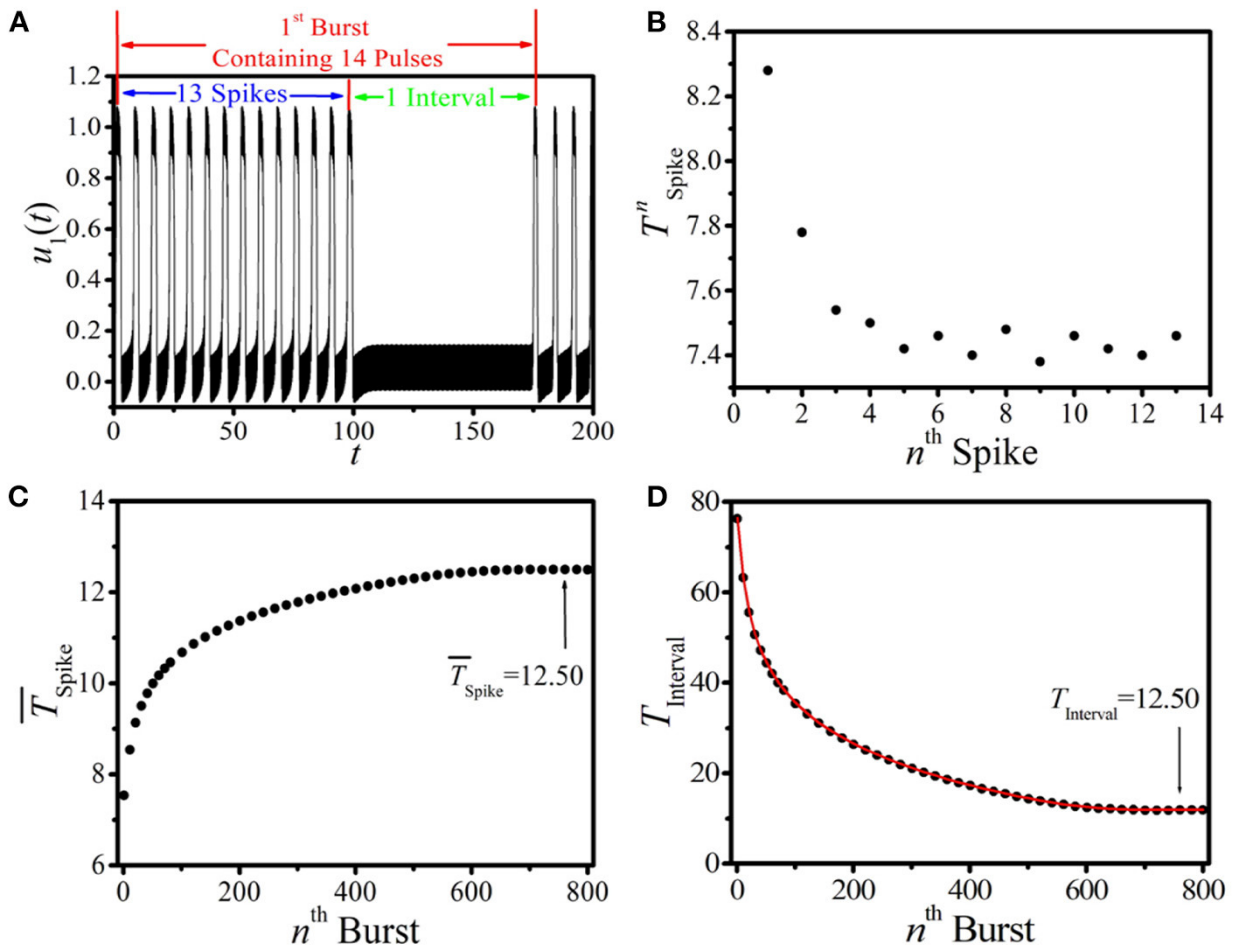
(Color online) **(A)** The illustration of the 1*st* burst period of the BOM pattern of Figure 2D. **(B)** The oscillation periods of the *N*_Pulse_ − 1 short spikes $T_{\text{Spike}}^{n}$ of **(A)**. **(C,D)** The dependence of ${\bar{T}}_{\text{Spike}}$ **(C)** and *T*_Interval_ **(D)** of BOM on the sequence number of burst-oscillation. Red line and black dots in **(D)** correspond to theoretical prediction and numerical results, respectively.

It is important to analyze the dynamical features and the evolution of this new type of BOM for a given linking time. Here, we choose the BOM pattern at the linking time *T*_Link_ = 100 shown in Figure 2D as our example, and the analysis of the corresponding 1*st* burst period is displayed in Figure 3A. It is shown that there are *N*_Pulse_ = 14 pulses in one burst period, which consists of *N*_Pulse_ − 1 short spikes (named as the *spike phase*) and a long interval (called the *interval phase*). We first discuss the period of the spike phase of BOM, and the periods of each short spike in the 1*st* burst period of Figure 3A are displayed in Figure 3B, where the oscillation periods of these *N*_Pulse_ − 1 short spikes labeled by $T_{\text{Spike}}^{n}(n=1,2,\ldots ,N_{\text{Pulse}}-1)$ (*n* = 1, 2, …, *N*_Pulse_ − 1) are different. It is shown that as the short spike excited by the external periodic pacing evolves, the oscillation period of the corresponding *n*^*th*^ spike ${\bar{T}}_{\text{Spike}}=\frac{1}{N_{\text{Pulse}}-1}{\sum_{n=1}^{N_{\text{Pulse}}-1}T^{n}}_{\text{Spike}}$ decreases obviously and saturates to a stable value (around 7.46). To depict the period of the spike phase of BOM uniquely, let us introduce the average period of these *N*_Pulse_ − 1 short spikes in one burst period as $T_{\text{Spike}}^{n}$. Figure 3C gives the dependence of ${\bar{T}}_{\text{Spike}}$ on the sequence number of burst-oscillation, where ${\bar{T}}_{\text{Spike}}$ increases with the bursts and approaches a fixed value of about 12.50.

The duration of the interval phase of BOM *T*_Interval_ is another important quantity in describing the firing process. Let us define the time that the excitable wave needs to fulfill a complete propagation along the ring as *T*_Length_. For a given BOM, *T*_Length_ consists of the periods of the spike phase and the interval phase, which can be approximatively expressed as


(6) $$\begin{array}{l} T_{\text{Length}}\approx \left(N_{\text{Pulse}}-1\right)*{\bar{T}}_{\text{Spike}}+T_{\text{Interval}}. \end{array}$$

In this formula, we use the average period ${\bar{T}}_{\text{Spike}}$ to approximatively represent *N*_Pulse_ − 1 different spike periods in one burst period. Then, the interval time of BOM is


(7) $$\begin{array}{l} T_{\text{Interval}}\approx T_{\text{Length}}-\left(N_{\text{Pulse}}-1\right)*{\bar{T}}_{\text{Spike}}. \end{array}$$

This implies that the interval time of BOM *T*_Interval_ is related to *T*_Length_, *N*_Pulse_, and ${\bar{T}}_{\text{Spike}}$. Because of the fixed values of *T*_Length_ and *N*_Pulse_ at a certain set of parameters and the feature of ${\bar{T}}_{\text{Spike}}$ shown in Figure 3C, *T*_Interval_ at a specific linking time should decrease as the BOM evolves. Theoretical predictions according to Equation (7) (red curve) and numerical results (black dots) of *T*_Interval_ are plotted in Figure 3D, and they coincide well with each other. It can also be found that as BOM evolves *T*_Interval_ decreases remarkably and tends to the time that equals the long-term average period of the spike phase, i.e., *T*_Interval_=${\bar{T}}_{\text{Spike}}$=12.50. This means that the interval phase of BOM may vanish and the initial inhomogeneous BOM will eventually evolve to the homogeneous *spike-oscillation mode* (SOM) with uniform oscillation period. Therefore, the BOM in the 1D paced unidirectional excitable ring may be a transient state.

**Figure 4:**
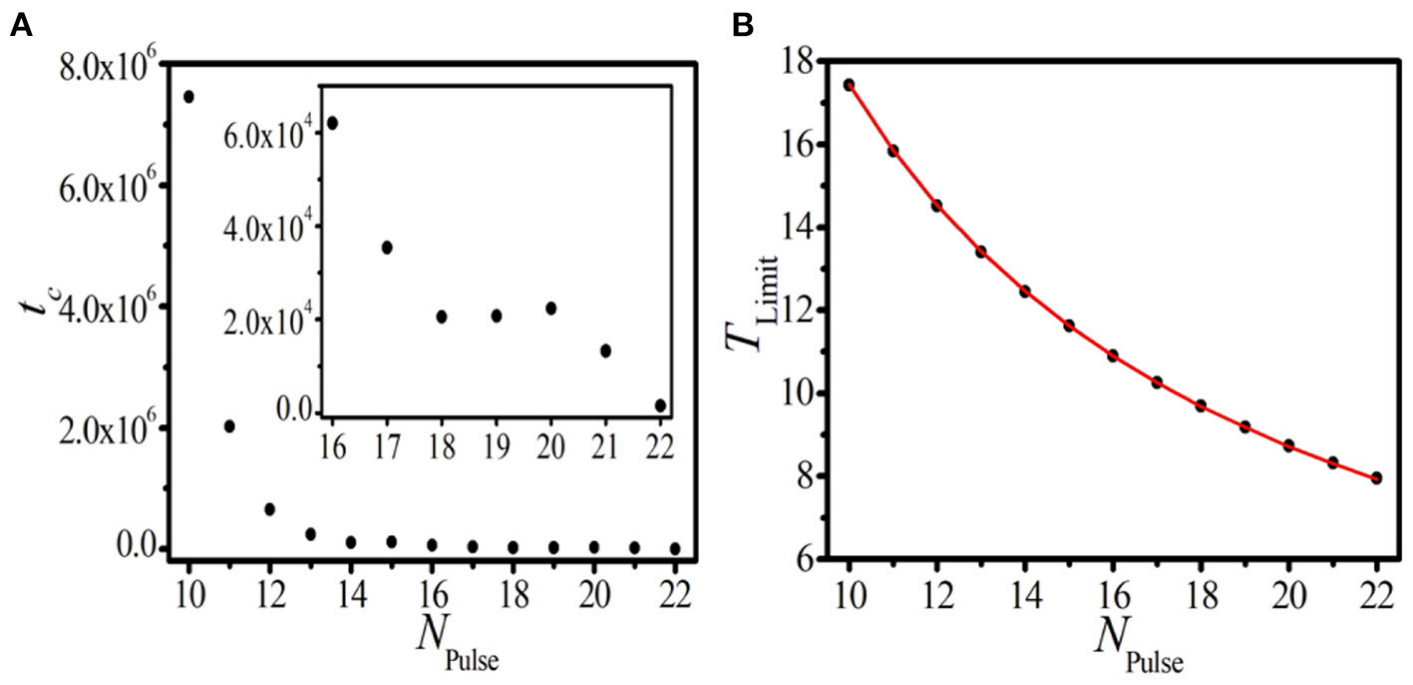
(Color online) The dependence of the relaxation time *t*_*c*_ **(A)** and the limit period of SOM *T*_Limit_ **(B)** on the initial pulse number of BOM *N*_Pulse_ in the realizable parameter region. The inset in **(A)** displays the local amplification in the region *N*_Pulse_ ∈ [16, 22]. Red line and black dots in **(B)** correspond to theoretical prediction and numerical results, respectively.

To test this conjecture, the relaxation time that the initial inhomogeneous BOM evolves to the homogeneous SOM *t*_*c*_ should be taken into account. In Figure 4A, the relationship between *t*_*c*_ and the initial pulse number of BOM *N*_Pulse_ is presented. The inset of Figure 4A displays the local amplification in the region *N*_Pulse_ ∈ [16, 22]. It can be found that *t*_*c*_ increases sharply as *N*_Pulse_ decreases gradually, implying that the fewer the initial pulse number is, the more time it takes for the BOM finally evolving to the SOM. Let us present some more detailed discussion of this topic. As shown in Figure 4A, for the pulse number *N*_Pulse_ = 22, it takes $t_{c}\approx 1.5\times 10^{3}$ time units (t.u.) for the BOM to realize the relaxation. When *N*_Pulse_ decreases to 16, *t*_*c*_ slightly increases to 6.2 × 10^4^ t.u. However, when to *N*_Pulse_ = 10, the initial inhomogeneous BOM needs about $t_{c}\approx 7.5\times 10^{6}$ t.u. to evolve to the SOM, which is much longer than the former cases. More importantly, for the case of *N*_Pulse_ < 10, the evolution time *t*_*c*_ tends to diverge to infinite. Therefore, two parameter regions for the BOM whether it can evolve to the SOM, i.e., the realizable region (*N*_Pulse_ ∈ [10, 22]) and the unrealizable region (*N*_Pulse_ < 10), can be identified. Consequently, we think that the new type of BOM revealed in this paper is not necessarily a transient state and is worth deeper discussions.

To further reveal the dynamical features of the homogeneous SOM, it is valuable to study the corresponding oscillation period of the SOM, which is called as the limit period *T*_Limit_. The dependence of *T*_Limit_ on *N*_Pulse_ in the realizable parameter region is displayed in Figure 4B, where red line and black dots correspond to theoretical prediction and numerical results, respectively. Based on the long-term uniform periods of the spike phase and the interval phase shown in Figures 3C,D, we can infer that, in the realizable region, the initial inhomogeneous BOM containing both the spike and interval phases will self-organize and evolve to the homogeneous SOM with an equal period. This means that the initial inhomogeneous *N*_Pulse_ pulses will be evenly distributed in the 1D paced unidirectional excitable ring in the end, which is definitely an interesting phenomenon. So, the limit period of SOM *T*_Limit_ should be determined by the initial pulse number of BOM *N*_Pulse_ and the propagation time that the excitable wave needs to travel through the ring denoted by *T*_Length_, and the predicted limit period in the realizable parameter region can be calculated according to


(8) $$\begin{array}{l} T_{\text{Limit}}=T_{\text{Length}}/N_{\text{Pulse}}. \end{array}$$

The coincidence between theoretical prediction and numerical results shown in Figure 4B confirms our conjecture. These results indicate that the initial pulse number in the 1D ring is not only a key factor to achieve different parameter regions for BOM, but also can determine the period of SOM in the realizable region. This gives us a clue to produce the homogeneous distributed SOM in the 1D paced unidirectional excitable ring with a given period, which may have useful applications in the related practical systems.

## The Determinants of BOM

The above discussions revealed that the pulse number *N*_Pulse_, the average period of the spike phase ${\bar{T}}_{\text{Spike}}$, and the interval time between two successive burst periods *T*_Interval_ are three main quantities that can effectively depict the pattern of BOM. Consequently, it is promising for us to reveal the determinants of BOM by investigating the factors influencing these three quantities.

### Linking Time

In Figure 2, the response BOM patterns for different linking times *T*_Link_ have been revealed. It is shown that, as *T*_Link_ increases, the pulse number *N*_Pulse_ in one burst period increases. On the contrary, the interval time between two successive burst periods *T*_Interval_ decreases remarkably. This indicates that the linking time plays a key role in determining the burst pattern, which is closely related to the pulse number *N*_Pulse_ and the interval time *T*_Interval_.

**Figure 5:**
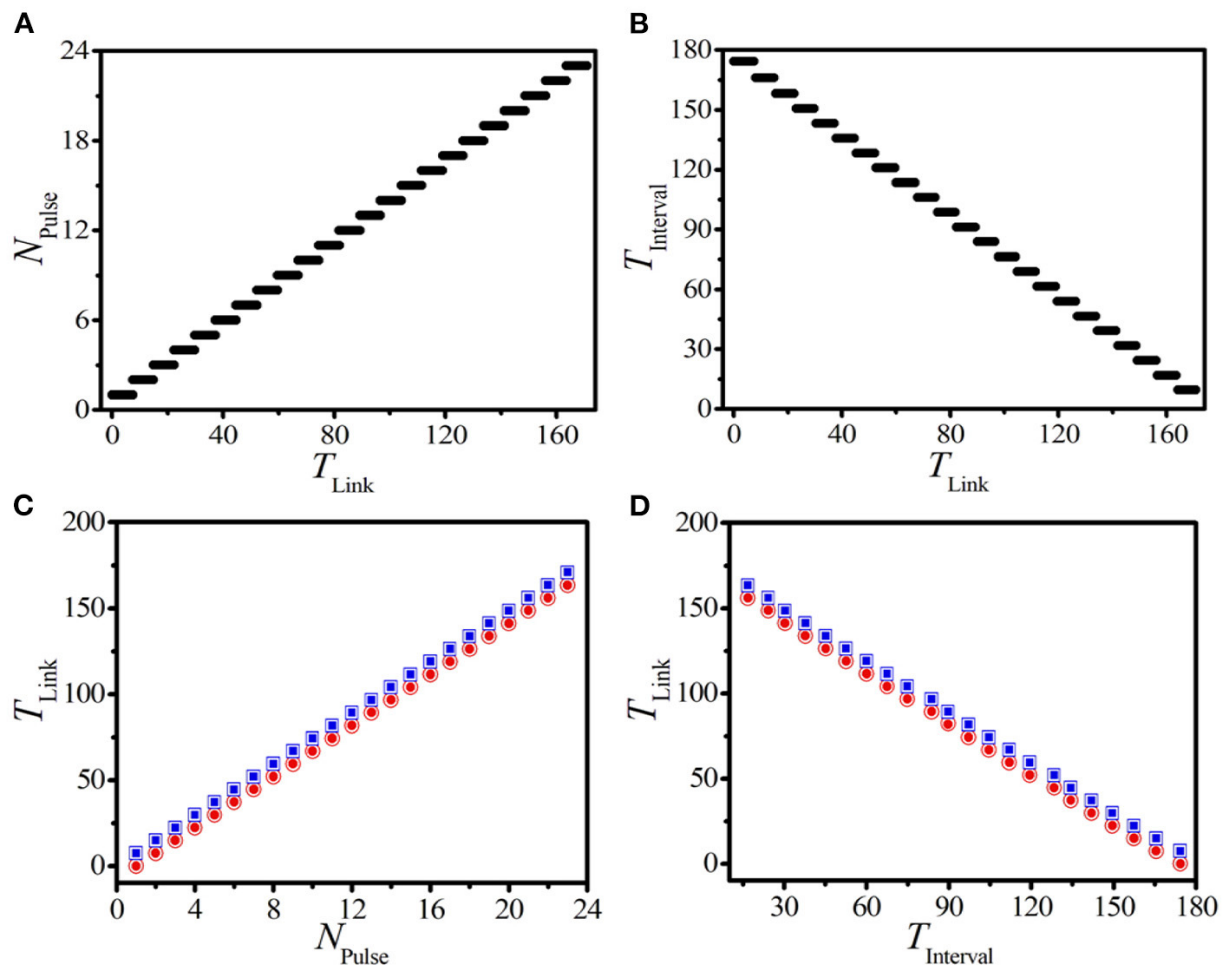
(Color online) **(A,B)** The dependence of the pulse number *N*_Pulse_ **(A)** and the interval time *T*_Interval_ **(B)** of BOM on the linking time *T*_Link_. Here, we should mention that the interval time *T*_Interval_ is measured from the 2*nd* burst period so as to avoid the influence from the initial external pacing. **(C,D)** The theoretical predictions (hollows) and numerical results (solids) of the lower (red circles) and upper (blue squares) thresholds of linking time vs. *N*_Pulse_ **(C)** and *T*_Interval_ **(D)**.

Figure 5A displays the relationship between the pulse number of BOM *N*_Pulse_ and the linking time *T*_Link_ on a 1D paced unidirectional excitable ring with the length *L* = 500 and the pacing parameters (*A,f*) = (2.5, 4.5). It is shown that *N*_Pulse_ increases stepwisely with the increase of *T*_Link_. Moreover, there exists a maximum pulse number for a given length of the 1D excitable ring. For example, $N_{\text{Pulse}}^{Max}=23$ for *L* = 500 shown in Figure 5A. The closer the pulse number *N*_Pulse_ approaches to its maximum value, the faster the initial inhomogeneous BOM evolves to the evenly distributed SOM. This interprets why the transition time *t*_*c*_ in the realizable parameter region closing to $N_{\text{Pulse}}^{Max}$ is very short (shown in Figure 4A).

Now, we discuss the influence of the linking time on the interval time of BOM. According to Equation (7) and the stepwise behavior of *N*_Pulse_ shown in Figure 5A, we can speculate that *T*_Interval_ should decrease in a similar stepwise manner for a certain set of system parameters. Figure 5B exhibits the dependence of *T*_Interval_ on *T*_Link_, where the stepwise decrease of *T*_Interval_ is exposed explicitly. This indicates that the more pulses created on the BOM, the fewer interval time will be obtained.

We can also infer from Figures 5A,B that, in each step of *N*_Pulse_ and *T*_Interval_, there exist a certain range of *T*_Link_ that can produce a specific BOM with the same pulse number and interval time. This indicates that there exists two critical values for the linking time to realize the same BOM pattern. Because the supra-threshold spiking will be suppressed as long as a unidirectional link from the end node *i* = *L* to the head node *i* = 1 is applied (shown in Figures 1A,C). So, the number of supra-threshold spiking pulses, which is retained in the process of constructing the 1D ring, is the key point to produce the BOM pattern with the same *N*_Pulse_. More importantly, due to the existence of the oscillation period of a single supra-threshold pulse, we can realize a specific BOM pattern with the same pulse number in a certain range of the linking time. Therefore, the lower and upper thresholds of the linking time that can realize the BOM with a given *N*_Pulse_ can be estimated as


(9) $$\begin{array}{l} T_{\text{Chain}}*\left(N_{\text{Pulse}}-1\right)<T_{\text{Link}}<T_{\text{Chain}}*N_{\text{Pulse}}. \end{array}$$

Here, *T*_Chain_ represents the response period of a single supra-threshold pulse in the unidirectional chain of Figure 1A. For a pacing with (*A,f*) = (2.5, 4.5), *T*_Chain_ ≈ 7.43. To test the validity of (9), the numerically lower and upper thresholds of *T*_Link_ are obtained based on the data shown in Figure 5A. Theoretical predictions (hollows) and numerical results (solids) of the lower (red circles) and upper (blue squares) thresholds of the linking time varying with the pulse number in Figure 5C clearly show the coincidence between theoretical predictions and experimental results.

We can also use the interval time of BOM to predict the two corresponding critical values of the linking time. By combining Equations (7) and (9), we can obtain the corresponding prediction formula of *T*_Link_ based on the given interval time of BOM *T*_Interval_ as


(10) $$\begin{array}{l} \frac{T_{\text{Chain}}}{{\bar{T}}_{\text{Spike}}}\left(T_{\text{Length}}-T_{\text{Interval}}\right)<T_{\text{Link}}<\frac{T_{\text{Chain}}}{{\bar{T}}_{\text{Spike}}}\\ \left(T_{\text{Length}}-T_{\text{Interval}}+{\bar{T}}_{\text{Spike}}\right). \end{array}$$

Figure 5D displays the theoretical and numerical thresholds of *T*_Link_ based on the interval time *T*_Interval_. The consistency further confirms the above theoretical predictions. The results shown in Figure 5 indicates that the linking time is a key factor in determining the new type of BOM in the 1D paced unidirectional excitable ring. Importantly, the pattern of BOM can be accurately created by choosing a suitable linking time according to the given pulse number or interval time.

### System Length

**Figure 6:**
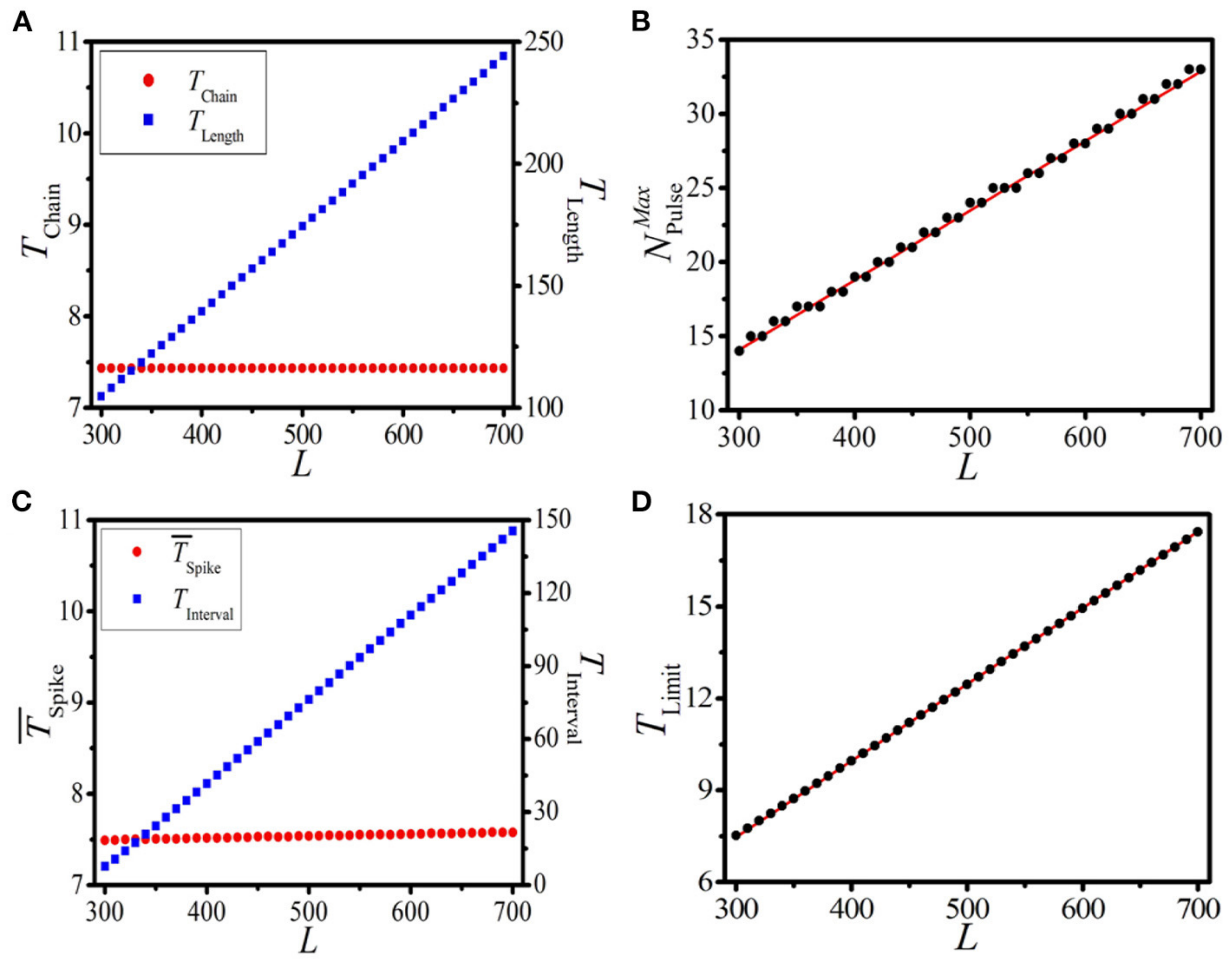
(Color online) **(A)** The dependence of *T*_Chain_ (left vertical axis) and *T*_Length_ (right vertical axis) on the system length *L*. **(B)** The theoretical prediction (red line) and numerical results (black dots) of the maximum pulse number $N_{\text{Pulse}}^{Max}$ vs. *L*. **(C,D)** The dependence of ${\bar{T}}_{\text{Spike}}$ [left vertical axis in **(C)**] and *T*_Interval_ [right vertical axis in **(C)**] of the BOM (measured from the 2*nd* burst period), and the theoretical prediction [red line in **(D)**] and numerical results [black dots in **(D)**] of *T*_Limit_ of the SOM on *L* with *N*_Pulse_ = 14.

Here, we study the effect of the system length *L* on the response dynamics of the 1D paced excitable system. According to Equations (7)-(11), we can find that *T*_Chain_ and *T*_Length_ are two key indices in determining the dynamical features of BOM. Let us first discuss the impacts of *L* on *T*_Chain_ and *T*_Length_, which are shown in Figure 6A, where the left and right vertical axes denote *T*_Chain_ and *T*_Length_, respectively. It is shown that as *L* increases from 300 to 700, *T*_Chain_ always keeps at 7.43, while *T*_Length_ increases from 104.65 to 244.19. This means that system length has no effect on the response period of a single supra-threshold pulse, while remarkably increasing the propagation time of an excitable wave along the ring.

Furthermore, for a given set of *T*_Chain_ and *T*_Length_, one can derive the maximum pulse number in the 1D ring $N_{\text{Pulse}}^{Max}$ as


(11) $$\begin{array}{l} N_{\text{Pulse}}^{Max}\approx \frac{T_{\text{Length}}}{T_{\text{Chain}}}. \end{array}$$

Based on Equation (11) and the *T*_Chain_ ~ *L* and *T*_Length_ ~ *L* relationships shown in Figure 6A, we can theoretically predict the relationship between $N_{\text{Pulse}}^{Max}$ and *L*. As displayed in Figure 6B, theoretical prediction (red line) and numerical results (black dots) of $N_{\text{Pulse}}^{Max}$ vs. *L* are presented. It is shown that as the system length *L* increases, the maximum pulse number $N_{\text{Pulse}}^{Max}$ increases stepwise. Clearly, the coincidence of theoretical prediction and numerical results further confirms the formula (9) of $N_{\text{Pulse}}^{Max}$.

It is necessary to discuss the influences of the system length *L* on the BOM (characterized by ${\bar{T}}_{\text{Spike}}$ and *T*_Interval_) and the SOM (characterized by *T*_Limit_). The pulse number *N*_Pulse_ = 14 is adopted as an example, which is located in the realizable region. Figure 6C displays the dependence of ${\bar{T}}_{\text{Spike}}$ (left vertical axis) and *T*_Interval_ (right vertical axis) on *L*. It is shown that ${\bar{T}}_{\text{Spike}}$ always keeps constant while *T*_Interval_ increases linearly with the increase of *L*. This means that, for the BOM, the system length *L* has no effect on the average period of the spike phase and has a linear relationship with the interval time. This coincides with our theoretical result given by Equation (7).

Figure 6D exhibits the theoretical prediction (red line) and numerical results (black dots) of *T*_Limit_ of the SOM on *L*. Perfect coincidence indicates that with a given initial pulse number, the oscillation period of SOM will increase as the system length *L* is increased. This well confirms that the system length is a key factor in determining the response dynamics of the 1D paced excitable system, including the propagation time of an excitable wave along the loop, the maximum pulse number, the interval time of BOM, and the limit period of SOM. All these key quantities exhibit linear relationships with the system length.

### Local Dynamics

**Figure 7:**
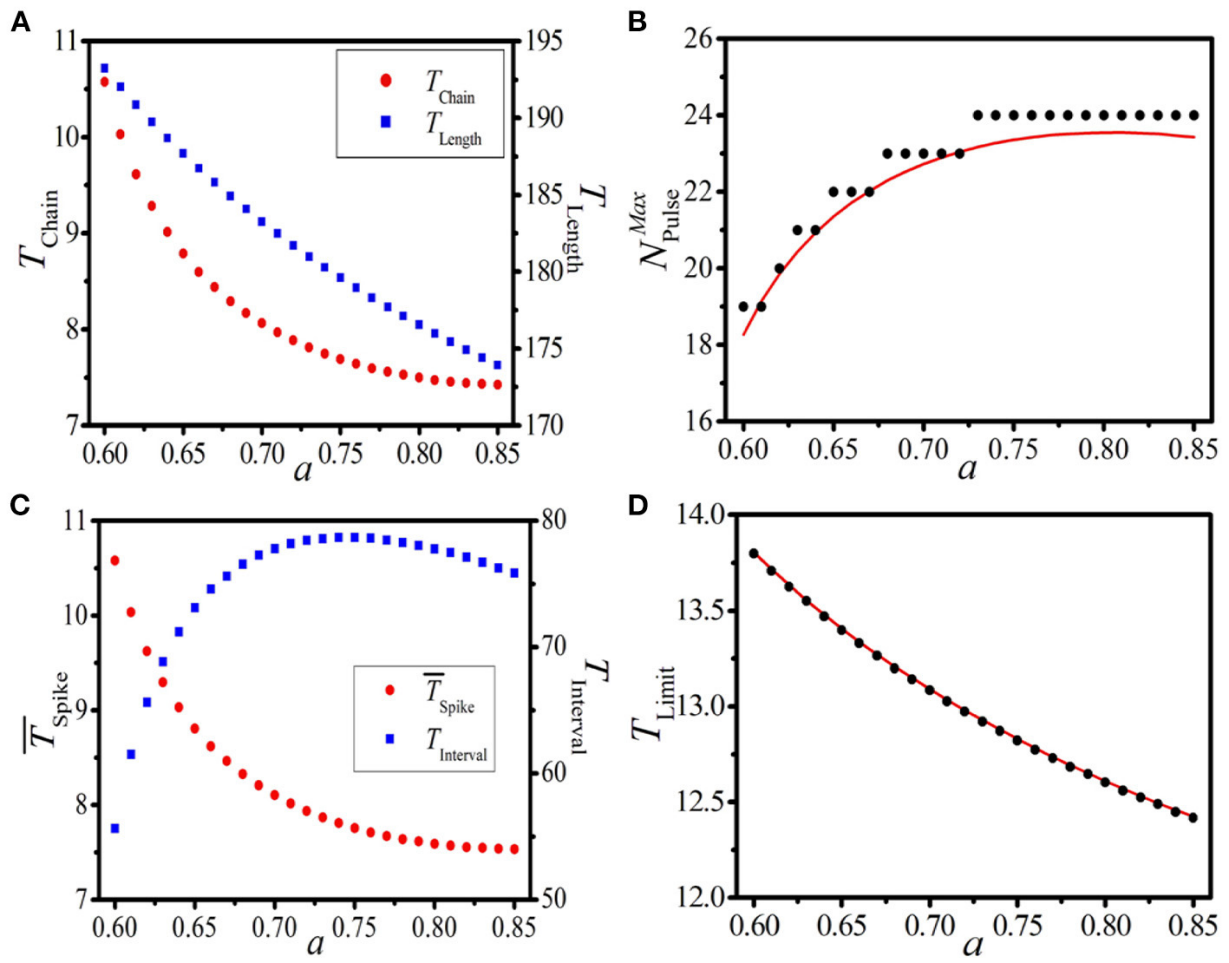
(Color online) **(A)**: The dependence of *T*_Chain_ (left vertical axis) and *T*_Length_ (right vertical axis) on the local dynamical parameter *a* with *L* = 500. **(B)**: The theoretical prediction (red line) and numerical results (black dots) of the maximum pulse number $N_{\text{Pulse}}^{Max}$ vs. *a*. **(C,D)**: The dependence of ${\bar{T}}_{\text{Spike}}$ [left vertical axis in **(C)**] and *T*_Interval_ [right vertical axis in **(C)**] of the BOM (measured from the 2*nd* burst period), and the theoretical prediction [red line in **(D)**] and numerical results [black dots in **(D)**] of *T*_Limit_ of the SOM on *a* with *N*_Pulse_ = 14.

Now, we discuss the impact of the local dynamics on the response behavior. We choose the dynamical parameter *a* as the testing parameter, and similar discussions as Figure 6 are carried out. Figure 7A displays the dependence of *T*_Chain_ (left vertical axis) and *T*_Length_ (right vertical axis) on the local dynamical parameter *a* with system length *L* = 500. It is shown that *T*_Chain_ and *T*_Length_ decrease remarkably with increasing *a*. Theoretical prediction (red line) and numerical results (black dots) of the maximum pulse number $N_{\text{Pulse}}^{Max}$ vs. *a* are presented in Figure 7B, where the stepwise increase of $N_{\text{Pulse}}^{Max}$ is exposed. Importantly, the larger the *a* is, the broader the step is. Based on the distinct variations of these three quantities induced by the local dynamical parameter *a*, the dramatic response dynamics of the BOM and the SOM can be expected.

To verify the above speculation, let us take the pulse number *N*_Pulse_ = 14 as an example. Figure 7C displays the dependence of ${\bar{T}}_{\text{Spike}}$ (left vertical axis) and *T*_Interval_ (right vertical axis) of the BOM on *a*. It is shown that the average period of the spike phase of BOM decreases with increasing *a*, and consequently the interval time of BOM increases. Figure 7D gives the theoretical prediction (red line) and numerical results (black dots) of the limit period of SOM on *a*, where *T*_Limit_ decreases with the increase of the local dynamical parameter. These results strongly indicate that the local dynamics is also a determinant of the response dynamics in the 1D paced unidirectional excitable ring, which can effectively control the patterns of BOM and SOM by regulating the dynamics of the local excitable model.

## The Parameter Region for BOM

It has been shown in Figure 1 that distinct responses in the 1D periodically paced unidirectional excitable chain and ring can be obtained with different pacings. It is important to study the external pacing as a key factor and the effective parameter region of external pacing in producing BOM. Furthermore, as shown in Figures 1C,D, the boundary condition is also very important, where the mode transition from the supra-threshold spiking to the sub-threshold vibration with different boundary conditions is a vital point to expose the effective pacing parameter region for BOM. Consequently, revealing the typical parameter regions of the supra-threshold spiking and the sub-threshold vibration with different boundary conditions will help us expose the available parameter combinations for BOM.

**Figure 8:**
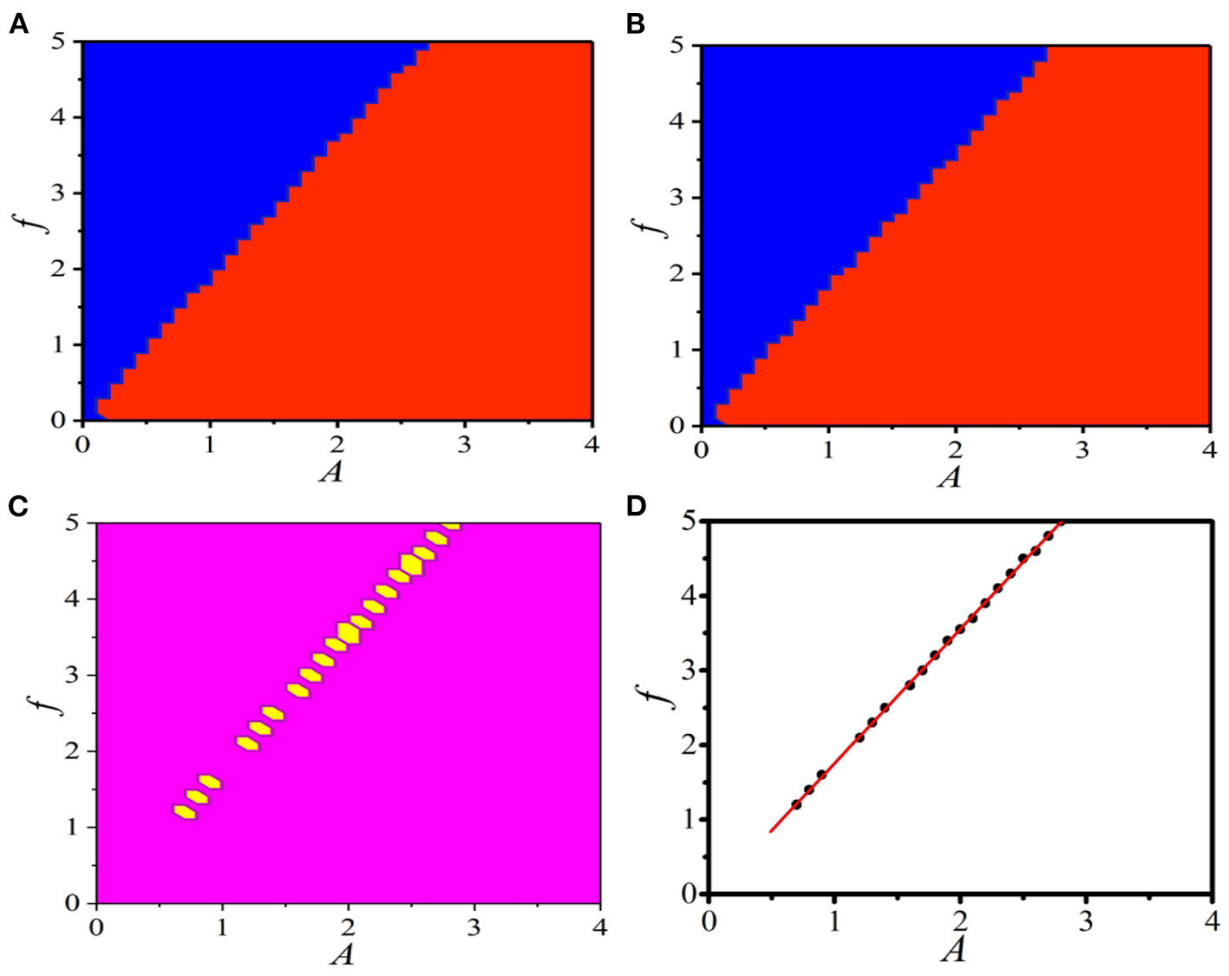
(Color online) **(A,B)** The distinct parameter regions in (*A,f*) plane for the supra-threshold spiking (red domains) and the sub-threshold vibration (blue domains) in the 1D paced unidirectional excitable ring with different boundary conditions: the periodic boundary condition *u*_*i*+*L*_(*t*) = *u*_*i*_(*t*), *v*_*i*+*L*_(*t*) = *v*_*i*_(*t*) **(A)** and the fixed boundary condition *u*(*L*) = *v*(*L*) = 0 **(B)**. **(C)** The same response dynamics regions (large pink regions) and the different response dynamics regions (small yellow hexagonal regions) for the periodic and the fixed boundary conditions. **(D)** Several parameter combinations of (*A, f*) (black dots) to produce the BOM. The linear fitting gives the relationship as *f* = −0.04 + 1.80 * *A* (red line).

Figure 8A displays the distinct parameter regions in (*A, f*) plane for the supra-threshold spiking and the sub-threshold vibration in the 1D paced unidirectional excitable ring with the periodic boundary condition *u*_*i*+*L*_(*t*) = *u*_*i*_(*t*), *v*_*i*+*L*_(*t*) = *v*_*i*_(*t*). It is shown that the parameter region of the supra-threshold spiking is larger (red domains) than that of the sub-threshold vibration (blue domains). For the fixed boundary condition *u*_*L*_(*t*) = *v*_*L*_(*t*) = 0, the phase diagram of (*A,f*) given in Figure 8B clearly shows that the distinct parameter regions of the supra-threshold spiking and the sub-threshold vibration have a slight shift. This comparison indicates that, as the boundary condition varies, the response dynamics with a specific parameter combinations of (*A,f*) will change, and the mode transition from the supra-threshold spiking to the sub-threshold vibration will occur.

To explicitly expose this interesting mode-transition region, Figure 8C gives the same response dynamics regions (large pink regions) and the different response dynamics regions (small yellow hexagonal regions). It is shown that the specific parameter region of oscillation mode transition does exists, and the new type of BOM can be realized in the 1D paced unidirectional excitable ring with these pacing parameter combinations. To quantitatively give the relationship between *A* and *f* in this effective parameter region for BOM, several combinations of (*A,f*) are studied, which are shown by the black dots in Figure 8D. Linear fitting gives the relationship as *f* = −0.04 + 1.80 * *A* (red line).

## Conclusion

In this paper, we extensively investigate the abundant response dynamics of the simplest 1D lattice of paced excitable systems with unidirectional coupling. Distinct oscillation modes, such as the supra-threshold spiking, the sub-threshold vibration, and the mode transition from the supra-threshold spiking to the sub-threshold vibration, have been observed by adjusting external pacings. Furthermore, a new type of BOM is revealed in the 1D unidirectional excitable ring for certain pacing parameters, which to our knowledge should be reported for the first time. It is shown that the BOM contains two distinct phases, i.e., the phase with multiple short spikes and the phase with a long time interval.

We further study the evolution of BOM, and two parameter regions, i.e., the realizable region and the unrealizable region are exposed, which is determined by the initial pulse number in the system. It is revealed that, in the realizable region, the initial inhomogeneous BOM will eventually evolve to the homogeneously distributed SOM, and the relaxation time *t*_*c*_ will decrease sharply as *N*_Pulse_ closes to its maximum value. This means that the more the initial pulse number is, the fewer relaxation time it takes for the BOM evolving to the SOM. However, in the unrealizable region, the BOM can still maintain. This confirms that the new type of BOM revealed in this paper is not necessarily a transient state and is worth further discussions. Consequently, several dynamical features of BOM and SOM, such as the average period of the spike phase ${\bar{T}}_{\text{Spike}}$, the interval time *T*_Interval_ of BOM, and the limit period of SOM *T*_Limit_, are theoretically predicted and have been verified in numerical simulations.

The mechanisms of the emergence of BOM are also studied in detail. It has been exposed that the linking time, the system length, and the local dynamics are three key factors that can effectively determine the dynamics of BOM. Specifically, the linking time *T*_Link_ can directly decide the pattern of BOM, especially the pulse number *N*_Pulse_ and the interval time *T*_Interval_. Importantly, the lower and the upper thresholds of the linking time, which can produce the given BOM, are theoretically predicted. The system length *L* can determine the dynamics of BOM by mainly impacting on the propagation time of an excitable wave along the loop *T*_Length_, the maximum pulse number $N_{\text{Pulse}}^{Max}$, the interval time of BOM *T*_Interval_, and the limit period of SOM *T*_Limit_. These four key quantities exhibit linear relationships with the length of the system. The local dynamical parameter can primarily influence the excitable dynamics of local cell, which will make a remarkable impact on the response dynamics of the 1D paced excitable systems. Consequently, the dynamical features of BOM and SOM will be affected obviously as the local parameter varies. Finally, the pacing parameter regions of (*A,f*), which can effectively produce the new type of BOM, have been explicitly exposed, and the relationship between *A* and *f* in this significant domain is quantitatively given.

Nowadays, the issue of burst oscillation in nonlinear science is a hot topic under investigation due to its extensive applications in a wide variety of natural systems. Different from the well-known bursts in single dynamical systems with distinct timescales, which are induced by the complex interactions between local fast and slow variables, we have proposed a network method to produce a new mode of burst oscillation in excitable complex networks. This kind of new BOM can keep the original perfect spiking dynamics of the local excitable system, and be easily regulated by manipulating the related key factors. This means that we can produce the BOM with specific dynamical features we wanted in real situations, especially the pulse number, the period of the spike phase, and the interval time, which are the main characteristic determinants of burst oscillation. It overcomes the difficulty that the mode of burst oscillation in a usual single dynamical system is hard to regulate and may have useful applications in practical systems in biology, chemistry, and physics. Our research extends the diversity of bursting types and provides more ways to bursting dynamics. We hope our contribution can shed light on a deeper understanding of bursts in nature and will have a useful impact in related fields.

## Data Availability Statement

The original contributions presented in the study are included in the article/supplementary material, further inquiries can be directed to the corresponding authors.

## Author Contributions

ZZ, YQ, ZL, and FL contributed to conception and design of the study. ZZ and YQ organized the database. ZL, YZ, and JL performed the statistical analysis. YQ wrote the first draft of the manuscript. ZZ and YQ wrote sections of the manuscript. All authors contributed to manuscript revision, read, and approved the submitted version.

## Funding

This work is supported by the National Natural Science Foundation of China (grant no. 11875135) and the Natural Science Basic Research Plan in Shaanxi Province of China (grant nos. 2022JZ-03, 2021JQ-811).

## Conflict of Interest

The authors declare that the research was conducted in the absence of any commercial or financial relationships that could be construed as a potential conflict of interest.

## Publisher's Note

All claims expressed in this article are solely those of the authors and do not necessarily represent those of their affiliated organizations, or those of the publisher, the editors and the reviewers. Any product that may be evaluated in this article, or claim that may be made by its manufacturer, is not guaranteed or endorsed by the publisher.
